# Detection and Classification of Saffron Adulterants by Vis-Nir Imaging, Chemical Analysis, and Soft Computing

**DOI:** 10.3390/foods12112192

**Published:** 2023-05-30

**Authors:** Pejman Alighaleh, Reyhaneh Pakdel, Narges Ghanei Ghooshkhaneh, Soodabeh Einafshar, Abbas Rohani, Mohammad Hossein Saeidirad

**Affiliations:** 1 Department of Biosystems Engineering, Faculty of Agriculture, Ferdowsi University of Mashhad, Mashhad P.O. Box 9177948974, Iran; pejman.alighaleh@gmail.com (P.A.); re.pakdel@mail.um.ac.ir (R.P.); narges.ghanei@gmail.com (N.G.G.); 2 Department of Agricultural Engineering Institute, Khorasan Razavi Agricultural and Natural Resources Research and Education Center, AREEO, Mashhad P.O. Box 9177335488, Iran; saiedirad@yahoo.com

**Keywords:** adulteration, classifiers, RGB images, saffron features, spectral images

## Abstract

Saffron (*Crocus sativus* L.) is the most expensive spice in the world, known for its unique aroma and coloring in the food industry. Hence, its high price is frequently adulterated. In the current study, a variety of soft computing methods, including classifiers (i.e., RBF, MLP, KNN, SVM, SOM, and LVQ), were employed to classify four samples of fake saffron (dyed citrus blossom, safflower, dyed fibers, and mixed stigma with stamens) and three samples of genuine saffron (dried by different methods). RGB and spectral images (near-infrared and red bands) were captured from prepared samples for analysis. The amount of crocin, safranal, and picrocrocin were measured chemically to compare the images’ analysis results. The comparison results of the classifiers indicated that KNN could classify RGB and NIR images of samples in the training phase with 100% accuracy. However, KNN’s accuracy for different samples in the test phase was between 71.31% and 88.10%. The RBF neural network achieved the highest accuracy in training, test, and total phases. The accuracy of 99.52% and 94.74% was obtained using the features extracted from RGB and spectral images, respectively. So, soft computing models are helpful tools for detecting and classifying fake and genuine saffron based on RGB and spectral images.

## 1. Introduction

Saffron (*Crocus sativus* L.), known for its unique aroma and coloring in the food industry, is the most expensive spice in the world [1,2]. Saffron is currently being cultivated in Iran, Spain, Italy, France, Turkey, Morocco, Japan, Israel, United Arab Emirates, China, Greece, India, Egypt, Switzerland, Azerbaijan, and Pakistan [3]. In 2018, the total world production of saffron was nearly 450 tons; 90% (approximately 404 tons) of that was produced in Iran [4]. Saffron is frequently adulterated. Sometimes other flowers of plants such as safflower (Carthamus tinctorius), marigold (Calendula officinalis), and arnica are fraudulently mixed with genuine saffron [3].

Various analytical methods are used to detect saffron quality and purity, including gas chromatography [5], thin layer chromatography (TLC) [6], liquid chromatography-mass spectroscopy (LC-MS) [7], nuclear magnetic resonance [8], and molecular methods (PCR) [9]. Despite having high accuracy and sensitivity, not only are these methods time-consuming and costly, but also they require specialist operators [10].

With recent advances in machine vision in terms of accuracy, robustness, and affordability, this technology has become suitable for determining the quality of saffron [11]. For example, Aghaei et al. [12] used machine vision technology to evaluate different saffron drying methods. Mohamadzadeh Moghadam et al. [11] used machine vision to classify different parts of saffron stigma (Pushal, Negin, and Sargol). Minaei et al. [13] used different color spaces to classify 33 samples of saffron from different geographical regions of Iran.

In the latest studies in saffron adulteration, machine vision systems based on deep learning were used. Deep learning, with its robust applicability, is increasingly prevalent due to rapid technological improvement [14]. Alighaleh et al. [15] used 10 convolutional networks to classify fake and genuine saffron. In the next study, six different categories of fake saffron were created, and a Learning-to-Augment incorporated Inception-v4 CNN was developed for grading and adulterants detection. The proposed LAII-v4 CNN achieved an accuracy of 99.5% [16]. Using deep learning methods has some drawbacks. One of the main challenges with deep learning is the need for a large amount of high-quality training data to produce accurate results [17].

Given that conventional imaging systems cannot identify specimens with similar colors, the spectroscopy technique can be a promising method for determining the quality of agricultural products based on the measurement of optical properties [18]. For instance, D’Archivio and Maggi [19] used the UV–visible spectroscopy technique to classify 81 geographically different samples of Italian saffron. In another study, NIR spectroscopy was used to determine the crocin of saffron samples obtained from Iran, China, Spain, Morocco, Nepal, and Greece [20]. In the subsequent study, two different adulterants (lotus stamens and corn stigmas) in saffron were identified and quantified using near-infrared spectroscopy [21].

While NIR spectroscopy only provides a mean spectrum of a sample, regardless of the area of the sample scanned [22], spectral imaging can be used to quantitatively predict a sample’s inherent chemical and physical properties and spatial distribution simultaneously [18]. In this case, features of images that can separate classes are extracted and used to classify genuine and fake saffron. Soft computing, such as neural networks, helps classify genuine and fake saffron more accurately.

Various algorithms such as K-nearest neighbor (KNN), support vector machine (SVM), self-organizing maps (SOM), multilayer perceptron (MLP), radial basis function neural network (RBF), learning vector quantization (LVQ), principal component analysis (PCA), etc., are used for classification. A wide range of research has been done employing these algorithms, such as the classification of rice grains [23] and wheat cultivars [24] using MLP neural network; classification of plant species using SVM [25]; classification of the dikarya fungi using SVM, KNN, and SOM [26]; sorting different parts of saffron with 22 different classifiers (SVM and KNN models) [11]; and classification of saffron and fake samples using PCA and PLS [27].

Due to the limited research conducted on the classification of saffron using visible and spectral imaging, the current study aimed to use and compare these two imaging methods in classifying images of genuine saffron and fake items (mixed stamen and dyed straw, dyed citrus blossom, safflower, and dyed fibers). In this paper, six classification methods, including SVM, SOM, MLP, KNN, LVQ, and RBF, have been used to detect and classify fake saffron from a genuine one.

## 2. Experiment

### 2.1. Sample Preparation

This study used seven different samples (four types of fake saffron andthree types of genuine saffron). Fake saffron samples included i. mixed stamen and dyed straw, ii. dyed citrus blossom, iii. safflower, and iv. dyed fibers. In this study, fake saffron samples were bought from the market. The genuine saffron was also collected from Zaveh county (located in Khorasan Razavi province, Iran). The genuine saffron samples were dried by three different methods of freeze drying, microwave drying, and hot-air drying to investigate the effect of drying methods on the quality and chemical content of saffron.

### 2.2. RGB Images Acquisition

**Figure 1:**
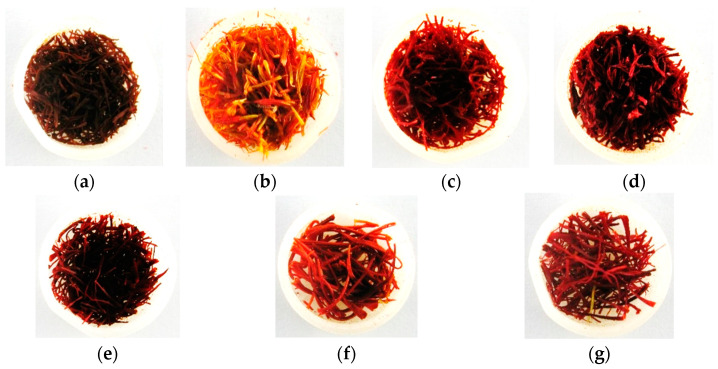
Sample of (**a**) dyed citrus blossom; (**b**) safflower; (**c**) mixed stamen and dyed straw; (**d**) dyed fibers; (**e**) microwave-dried saffron; (**f**) freeze-dried saffron; and (**g**) hot-air-dried saffron.

RGB imaging was done with a digital camera (Canon PowerShot G9, Made in Tokyo, Japan, 12MP) in December 2020. The samples were placed in a plastic 20 mm petri dish for imaging, and the distance between the camera lens and the samples was 10 cm. It should be noted that the imaging was performed in a specialized photo chamber with natural light between 10 and 12 P.M., without any light restrictions. Images were captured at their maximum resolution (3000 × 4000 pixels) and were saved in “JPG” format. The prepared dataset included a total number of 420 images in seven classes. It included 60 images for each class (Figure 1).

#### 2.2.1. Image Segmentation

**Figure 2:**
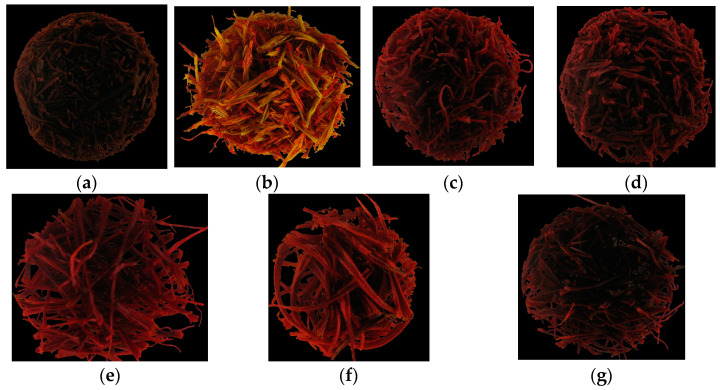
Sample of segmented images of (**a**) dyed citrus blossom; (**b**) safflower; (**c**) mixed stamen and dyed straw; (**d**) dyed fibers; (**e**) microwave-dried saffron; (**f**) freeze-dried saffron; and (**g**) hot-air-dried saffron.

After image acquisition, the data of each image must be extracted. Segmentation, hence, is an essential step to extract data [28]. Segmentation has two main parts: separating objects from the background and grouping related pixels together to form connected objects as regions of interest (ROI) [29]. Since ROI data are essential, noises of segmented images should be minimized. To extract the essential data and eliminate noises, images were segmented using the Otsu method (Figure 2).

#### 2.2.2. Extraction of Color Components

Color spaces that are frequently used include RGB (red, green, and blue), HSI (hue, saturation, and intensity), and L*a*b* (luminance, the red-green axis, and the blue-yellow axis); the transformation functions from RGB can be used to obtain any of these color space models [30]. Table 1 indicates the used color spaces and their channels, as well as the transformation functions of RGB. The mean and standard deviation for each color component, including R, G, B, H, S, I, L*, a*, and b*, were calculated in this study. To extract each of the color components mean, firstly, a binary image was multiplied by its color image, then the sum of values of the resulting matrix was divided into the number of binary image pixels. In total, considering the mean and standard deviation as two different features for each color component, 18 color features were extracted from each image.

#### 2.2.3. Texture Features

One of the most important characteristics that are used in image processing is texture analysis [30]. It is used to measure some properties of a region of a digital image, such as smoothness, uniformity, entropy, and so on [31,32].

Woods et al. [32] extracted three texture features—smoothness, uniformity, and entropy—from each image. These features are defined by various histogram indicators of an image, including mean and standard deviation, and can be calculated using Equations (1)–(3). Equation (1) measures the relative smoothness of the intensity in a region. *R* is 0 for a region of constant intensity and approaches 1 for the region with large variations in the values of its intensity levels [32].

(1) $$R=1-\frac{1}{\left(1+\sigma^{2}\right)}$$

**Figure 3:**
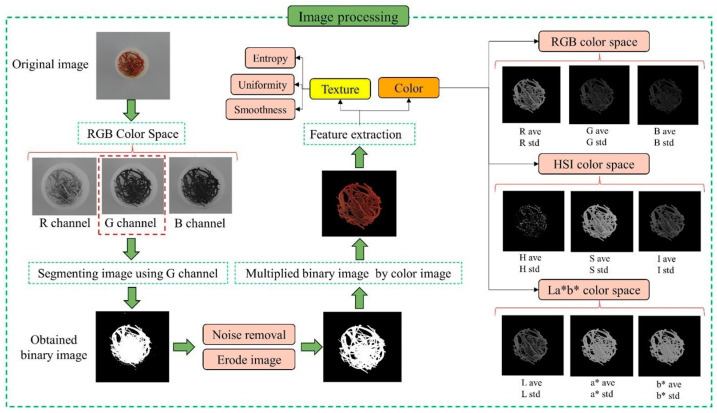
The process of image segmentation and feature extraction from RGB.

The variance, *σ^2^*, used in this measure is normalized to the range [0, 1]. Uniformity is calculated using Equation (2); this measure is maximum when all intensity values are equal (maximally uniform) and decreases from there. Equation (3) calculates entropy; entropy is a measure of disorder or randomness. Hence, the entropy will be greater for the rough texture because the distribution of pixel values in this area is more random.

(2) $$U=\overset{L-1}{\underset{i=0}{\sum }}p^{2}\left(z_{i}\right)$$

(3) $$e=-\overset{L-1}{\underset{i=0}{\sum }}p\left(z_{i}\right)\log p\left(z_{i}\right)$$

where $z_{i}$ is a discrete random variable that indicates the intensity of gray levels in an image, $p(z_{i})$ and *L* are the intensity histogram of an area or image and the extent of possible intensity of gray level, respectively [32]. The process of segmentation and feature extraction from color images is given in Figure 3. According to Figure 3, the RGB color space and the G color channel were utilized to divide the image into two parts: the background and the region of interest (ROI). After removing the noise and eroding on the segmented image, the resulting binary image was multiplied with the original RGB image. Then, the necessary information was extracted from the RGB image with a black background.

### 2.3. Spectral (Red and NIR) Imaging

Imaging in two bands, including near-infrared (NIR) (850 nanometers) and red (650 nanometers), was done by MAPIR Survey 2 camera (MAPIR company, made in the San Diego, CA, USA, 16MP). In this stage of imaging, a specialized photo chamber was used to reduce light noise. Due to the high lighting efficiency of tungsten halogen lamps within the NIR range of the electromagnetic spectrum [33], two lamps (GU 10, 50 W) were installed vertically on top of the light chamber. After imaging, to remove the effects of lamp lighting variations through calibration of raw images, a reference surface with 100% reflectance was used. From barium sulfate [34] and polytetrafluoroethylene (PTFE) [35] used in different studies as references, the latter was used in the current research. Through Equation (4), the calibrated image was obtained.

(4) $$R=\frac{R_{sample}-R_{dark}}{R_{reference}-R_{dark}}$$

where *R* is the calibrated sample image, *R_sample_* is the raw image, *R_reference_* is the image of the reference surface, *R_dark_* is the image of the reference surface with all lights out [33].

**Figure 4:**
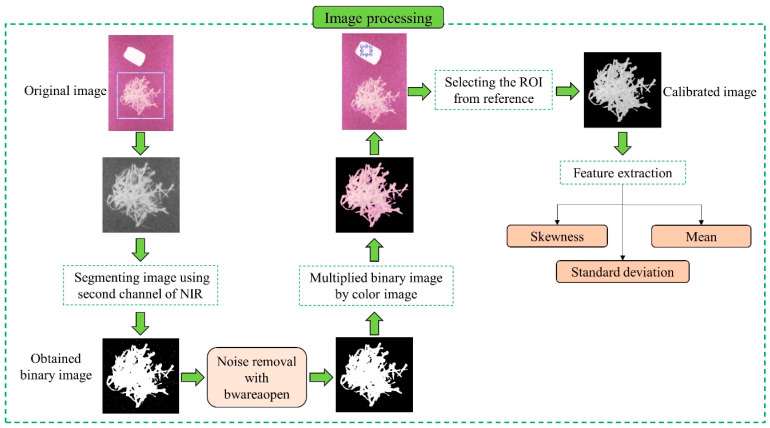
The process of image segmentation and feature extraction from spectral images.

After calibration, images were segmented and then the statistical parameters (such as mean, standard deviation, and skewness) of reluctance intensity at 850 and 650 nm were extracted to classify samples (Figure 4).

### 2.4. Chemical Analysis

According to the International Standard Organization method, ISO-3632 (2010), crocin, safranal, and picrocrocin of genuine and fake saffron were determined and standardized. In the first step, all samples were ground into a powder, then 500 mg of ground saffron was placed into a 1000 mL balloon, and about 900 mL of distilled water was added and stirred thoroughly. In the next step, the contents of the balloon were then shaken in a completely dark condition (the balloon was covered with aluminum foil) by a magnetic stirrer (LT108, V. 220, HZ. 50, Iran) at 1000 rpm for one hour. The content of the balloon was stirred to obtain a uniform solution. Then, 20 mL of the solution was transferred by pipette to a 200 mL flask and made up to the volume with distilled water. To obtain a clear solution, the prepared solution was filtered away from light using a strainer under a vacuum (Value, Model VE115N). The spectrometer (Spectronic unicam Genesysim8, Made in the Pittsburgh, PA, USA) was adjusted to 440, 330, and 257 nm wavelengths. The amounts of crocin, safranal, and picrocrocin were calculated using Equation (5):

(5) $$A_{1cm}^{1\%}\;\left(\lambda_{max}\right)=\frac{A\times 10,000}{0.5\times \left(100-H\right)}$$

where $A_{1cm}^{1\%}$ (257 nm) indicates maximum absorbance at 257 nm for Safranal, $A_{1cm}^{1\%}$ (330 nm) shows maximum absorbance at 330 nm for Picrocrocin, and $A_{1cm}^{1\%}$ (440 nm) indicates maximum absorbance at 440 nm for Crocin, *H*: humidity of 500 mg saffron.

### 2.5. Classifier Models

#### 2.5.1. Neural Networks

Neural networks are professional methods of classification and clustering. The LVQ neural network structure is based on dividing the input space R^n^ into several distinct regions, called decision regions, and for each region, one codebook vector is assigned. Classification is done based on the vicinity of the input vector x to the codebook vectors [36].

The SOM algorithm uses a competitive learning method for training and converts nonlinear statistical relationships between input data into simple geometric relationships [37]. In SOM, each neuron of the input layer (x) with a variable associated n-dimensional weight (w_ij_) is connected to all Kohonen neurons, and neurons are connected together by a neighborhood function [26,37].

MLP is a feed-forward layered network that has been constructed with one input layer, one output layer, and some hidden layers [38]. The sigmoid transfer function in the hidden layer, which can make the simple neural network suitable for estimation of any complex function, is determined based on Equation (6) [38]:

(6) $$f\left(\theta \right)=\frac{1}{1+e^{-\theta }}$$

Finally, the RBF neural network has a simple structure and fast learning algorithm compared with the other neural networks [39]. The input layer contains the properties extracted from images. The hidden layer of neurons is made up of a nonlinear function called radial base function or Gaussian function. RBF produces a linear output layer in the last step and that calculates the relationship between input and output [37]. In this study, several training algorithms were applied for MLP and RBF models, and the best one was selected by statistical analysis. Table 2 indicates the list of used training algorithms.

#### 2.5.2. Support Vector Machine (SVM)

SVM is a popular robust learning method [30]. It is a parametric statistical linear classifier that can map a nonlinear input space to a new linear feature space. SVM avoids overfitting by selecting the hyperplane with the maximum margins in feature space with an optimum separation [40]. The margin named as support vector is described as the minimal distance between the hyperplane and the boundary training examples [39,41].

#### 2.5.3. K-Nearest Neighbor Classifier (KNN) Algorithm

KNN is the simplest classification algorithm and works in two stages: training and testing stages [42]. Each pattern of training data set is represented in an N-dimensional space of the features during the training phase which is used to classify a new object. The K-nearest neighbor training patterns for each test pattern are determined using the distance function [26,43].

#### 2.5.4. Evaluation of Classification Performance

This study aimed to identify the genuine saffron and fake samples and classify them by mentioned classifiers. In this regard, accuracy (*ACC*) has been used to evaluate these models. The accuracy metric is the ratio of correct predictions over the total number of evaluated patterns, which is calculated using Equation (7):

(7) $$ACC=\frac{n_{p}}{n_{p}+n_{mp}}\times 100$$

where *n_p_* is the number of correctly classified samples and *n_mp_* the number of misclassified samples. It should be noted that the best classifier performance is achieved when the accuracy is close to a hundred [44].

### 2.6. Software

All image analyses, image calibrations, statistical analysis, modeling, and classification were performed in a custom-written MATLAB (R2019b, Mathworks, Inc., Natick, MA, USA) program.

## 3. Results and Discussion

### 3.1. The Results of Chemical Analysis

**Figure 5:**
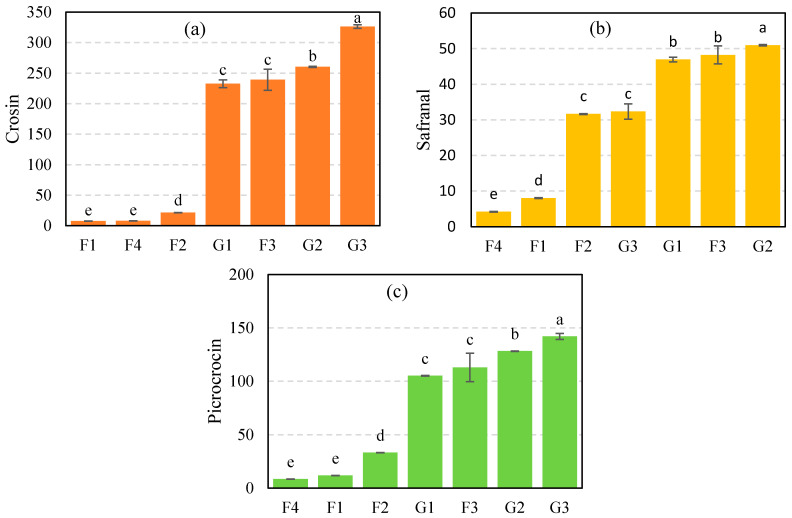
The mean comparison results for (**a**) crocin, (**b**) safranal, and (**c**) picrocrocin of samples by LSD method at the 5% significance level (Means that do not share a letter are significantly different). F1: dyed citrus blossom, F2: Safflower, F3: mixed stamen and dyed straw, F4: dyed fibers, G1: microwave-dried saffron, G2: freeze-dried saffron, and G3: hot-air-dried saffron.

The mean of crocin, safranal, and picrocrocin of samples was compared using the LSD method at a 5% significance level (Figure 5). According to ISO 3632-1, the mean of crocin, picrocrocin, and safranal in genuine saffron is more than 120, 40, and between 20 and 50, respectively [45]. As it is depicted, the mean crocin (Figure 5a), safranal (Figure 5b), and picrocrocin (Figure 5c) of three fake classes, including dyed citrus blossom (F1), safflower (F2), and fiber (F4) were significantly lower than other classes. In contrast, the chemical properties of mixed stamen and dyed straw (F3) were close to the genuine saffron classes. Then, the difference in the mean of crocin, safranal, and picrocrocin between F1 and microwave dried saffron (G1) was not significant. In addition, these results confirm that the mean of crocin, safranal, and picrocrocin is significantly dependent on drying methods. To be precise, the mean of crocin and picrocrocin in hot-air dried saffron (G3) is significantly higher than in other classes. Additionally, among diverse types of genuine saffron, freeze-dried (G2) and hot-air dried saffron (G3) had the highest and lowest amount of safranal, respectively.

### 3.2. Detection and Classification Using Vis-NIR Spectral Imaging

**Figure 6:**
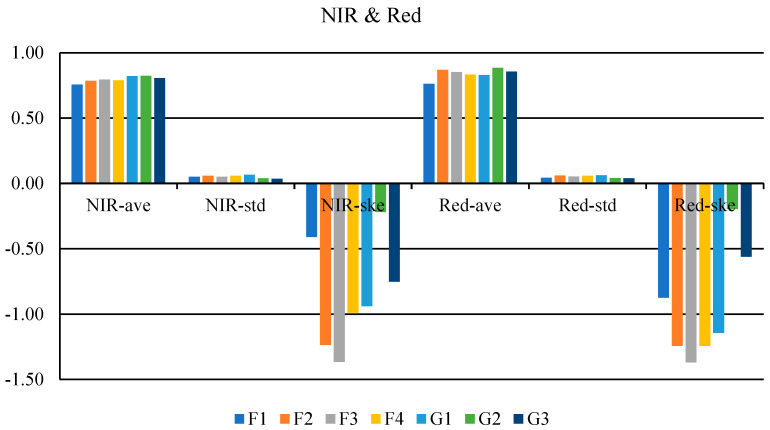
Comparing the mean statistical characteristics of Red (660 nm) and NIR (850 nm) spectra images using the LSD method. F1: dyed citrus blossom, F2: safflower, F3: mixed stamen and dyed straw, F4: dyed fibers, G1: microwave-dried saffron, G2: freeze-dried saffron, and G3: hot-air-dried saffron.

The results of comparing the mean, standard deviation, and skewness of NIR (850 nm) and Red (660 nm) spectra for seven classes of saffron by LSD method at 5% are shown in Figure 6. These results indicated that the calculated statistical characteristics could detect and classify genuine and fake saffron classes. In other words, the mean, standard deviation, and skewness of Red spectra were able to separate seven classes of saffron in 5, 3, and 5 classes, respectively. Additionally, NIR spectra images of samples were classified into 4, 4, and 5 classes based on the three parameters of mean, standard deviation, and skewness, respectively. Hence, these characteristics could be used as inputs for classifiers.

### 3.3. Detection and Classification Using RGB Imaging

**Figure 7:**
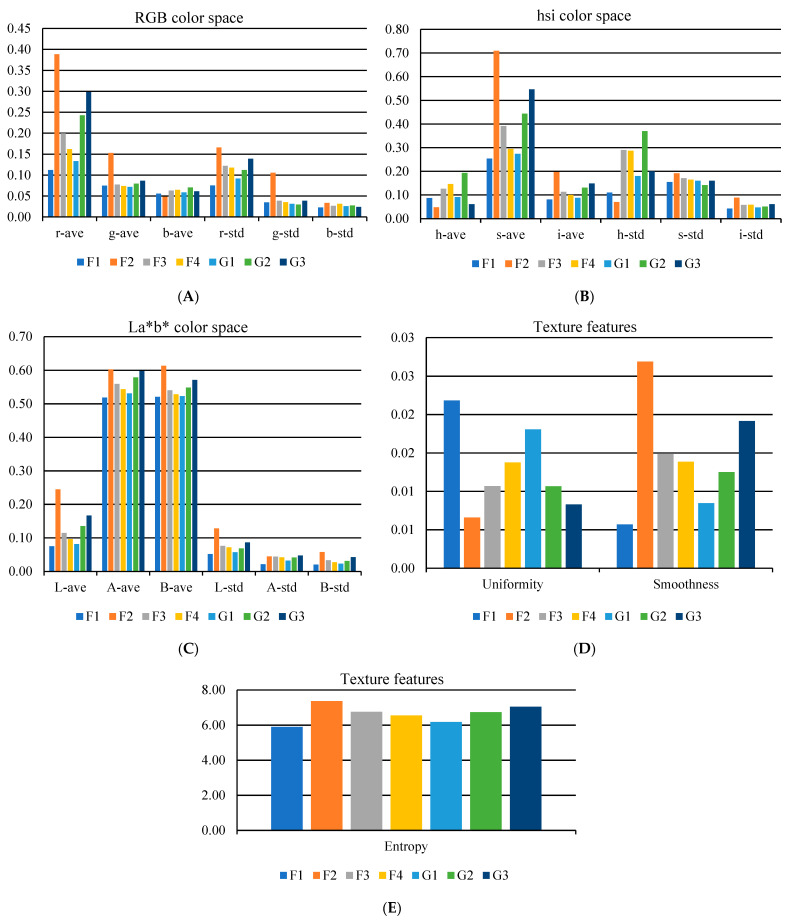
Comparing the mean and standard deviation of (**A**) RGB, (**B**) HSI, (**C**) L*a*b* and texture properties ((**D**) Uniformity and Smoothness, and (**E**) Entropy) of RGB images using the LSD method. F1: dyed citrus blossom, F2: safflower, F3: mixed stamen and dyed straw, F4: dyed fibers, G1: microwave-dried saffron, G2: freeze-dried saffron, and G3: hot-air-dried saffron.

Figure 7 compares the mean and standard deviation of color components, including R, G, B, H, S, I, L*, a*, b*, and texture properties extracted from RGB images. It should be noted that this comparison was done using the LSD method at a 5% level. As the figures show, all the extracted features can identify all samples. The importance of extracted properties will be evaluated using sensitivity analysis in the following.

### 3.4. Evaluation of Classifiers Performance

The accuracy of classifiers in three phases of training, testing, and total based on the extracted properties are shown in Table 3. These results confirm that the RBF neural network had a high ability to detect the type of saffron classes. In addition, three other classifiers, including MLP, KNN, and SVM, can be used in the next priorities because their detection accuracy in the test phase was lower than RBF.

Rezaei et al. [37] used KNN, SVM, SOM, and RBF neural networks to determine sex in immature pistachios. The results indicated that the KNN classifier was the most accurate (upper 95%) for sex determination and the next accurate classifier with 83.33% accuracy was RBF. In another study, Heidari et al. [46] used the morphological characteristics of pistachio leaves as the input data of the RBF neural network to identify pistachio kernel color with a 98.95% coefficient of determination. Another study confirmed that Camellia (Theaceae) Species could be classified using the SVM model with 97.92% accuracy [47].

### 3.5. Design and Evaluation of RBF Neural Network Based on Vis-NIR Results

**Figure 8:**
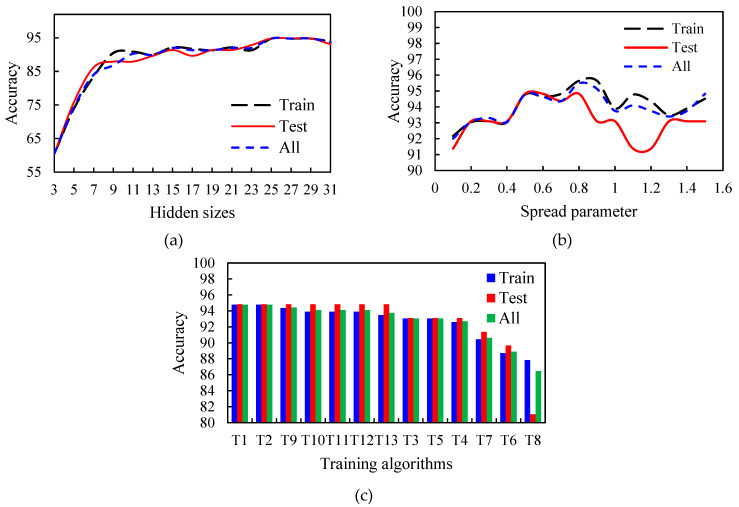
The effect of the number of neurons in the hidden layer (**a**), the spread parameter value (**b**), and the type of training algorithm (**c**) on the accuracy of the saffron classifier using Vis-NIR properties.

The number of neurons in the hidden layer, the spread parameter value, and the type of training algorithm are three important parameters required to design the RBF neural network. Figure 8 shows the effect of these parameters on the accuracy of the RBF algorithm in three different phases, including training, testing, and total. As demonstrated, the more number of neurons in the hidden layer, the more accuracy of saffron classification. To be precise, there was an upward trend from 3 to 25 neurons and then it became stable (Figure 8a). On the other hand, increasing the number of neurons raises the volume of calculations and training time. Hence, the number of neurons in the hidden layer was considered 25. Since the spread parameter (σ) can also affect the accuracy of the RBF algorithm, the effect of its value on this accuracy was investigated. Figure 8b indicates that increasing σ to near 0.9 led to a rise in the algorithm accuracy, and then there was a decrease. So, the optimum value of the spread parameter was considered 0.95. Another factor that can influence the selected classifier accuracy is the type of training algorithm. As Figure 8c shows, the two training algorithms, Levenberg–Marquardt (T1) and Bayesian Regularization (T2), had better performance in both training and testing phases. Additionally, they had higher accuracy than other training algorithms. By considering learning speed, the Levenberg–Marquardt (T1) training algorithm was selected.

The final results of the RBF neural network in the classification of four classes of fake saffron and three classes of genuine saffron are given in Table 4. As can be seen, although the overall accuracy of the RBF classifier was 94.79%, the detection accuracy of each class was different. For example, freeze-dried saffron was completely identified and classified. This was due to its different quality from the other two genuine classes of saffron. Therefore, it can be concluded that the better the quality of dried saffron, the more accurately it can be distinguished from fake saffron. This study showed that the use of Vis-NIR spectral features could not increase the detection accuracy by more than 94%. Therefore, to obtain higher accuracy, the features of RGB images should be investigated, which will be mentioned in the following sub-section. The results of the RBF neural network generalizability, based on Vis-NIR spectral features by decreasing the training data set and increasing the test data set, are given in Table 5. These results confirmed that a decrease in the size of the training set to about half of the total data set led to about 90% reduction of detection accuracy. Therefore, it can be claimed that the features of Vis-NIR images can be a good option for detecting fake saffron. On the other hand, this issue needs further investigation, especially at other wavelengths and a more complete data set. However, its generalizability can be acceptable under these conditions.

### 3.6. Design and Evaluation of RBF Neural Network Based on RGB Results

**Figure 9:**
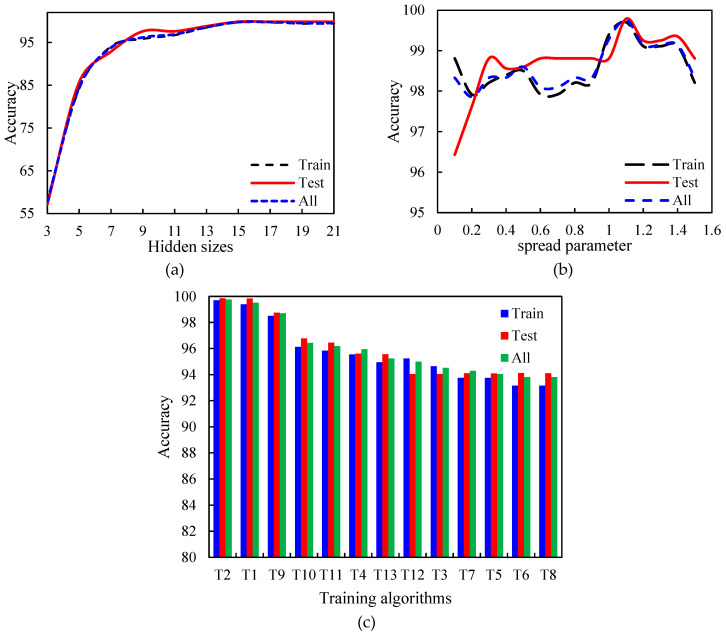
The effect of the number of neurons in the hidden layer (**a**), the spread parameter value (**b**), and the type of training algorithm (**c**) on the accuracy of the saffron classifier using RGB properties.

RGB image features were used (as mentioned earlier) as an accessible alternative solution to extract image features of seven classes of genuine and fake saffron and classify them. In addition, the RBF neural network classifier was selected from mentioned classifiers. To design this neural network, the number of neurons that have been used in the hidden layer was 17 (Figure 9a); and according to Figure 9b, the value of the spread parameter was 1. Although, Levenberg–Marquardt (T1) and Bayesian Regularization (T2) had better performance among other training algorithms (Figure 9c), the Bayesian Regularization (T2) algorithm was selected. 

A sensitivity analysis was carried out to determine the effectiveness of the extracted features of four groups, including HIS, RGB, L*a*b*, and texture. This analysis was done to reduce the number of features used in the neural network. Table 6 shows the sensitivity analysis results regarding the classification accuracy of different feature groups. As can be seen, when all 21 features were used as classifier input, the total accuracy was 99.52%. The results showed that when the features of the HSI group (h_ave, h_std, s_ave, s_std, i_ave, and i_std) were used, the highest accuracy (99.05%) was obtained in identifying and classifying samples. It also showed that the lowest accuracy (63.10%) was related to the texture features (smoothness, uniformity, and entropy). Finally, 14 features were selected from 21 features, including r_ave, b_ave, b_std, h_ave, s_ave, i_ave, h_std, l_ave, a_ave, b_ave, l_std, smoothness, uniformity, entropy using the trial-and-error method. Accordingly, the classification accuracy reached 99.52%, which was equal to the accuracy of the classifier using 21 features as input. Hence, the input vector size of the RBF model was reduced. The results of classification accuracy of the RBF classifier using 80% of the training data set and selected features are given in Table 7. As demonstrated, all classes’ detection and classification accuracy except F3 is equal to 100%.

Table 8 shows the results of the generalizability assessment of the RBF classifier by reducing the size of the training set from 80% to 50%. As the results reveal, although the reduction in the training data set’s size reduced the classification accuracy, the obtained 98.10% detection accuracy was still acceptable by using 50% of the training data set. In other words, the generalizability of the RBF classifier can be acceptable using 14 selected features.

### 3.7. Comparison with Similar Works

To evaluate the research, the results were compared with similar studies conducted to classify saffron types and detect saffron adulteration (Table 9). This comparison indicated that most of the methods are destructive and chemical, and destroy the samples and require special expertise and equipment. In addition, these methods are time-consuming despite their high accuracy. Hence, the present study used RGB and multispectral imaging methods to remove these drawbacks. The selected methods quickly detect and classify fake and genuine saffron with 99.52% accuracy without destroying the samples.

## 4. Conclusions

Given the challenges of accurately detecting saffron adulterations and the need for laboratory testing, which may not be accessible to many buyers and traders, the development of a non-destructive testing method could be a valuable solution. By using non-destructive testing methods, such as computer vision and machine learning algorithms, it is possible to analyze various features of saffron and detect any possible adulteration without damaging or destroying the product. In this article, machine learning methods were used to identify and classify four fake and three genuine saffron groups (dried with different methods). The results showed that chemical methods by measuring three chemical components of saffron (crocin, safranal, and picrocrocin) for detecting fake saffron and its quality were effective but time-consuming and costly. So, their application in the rapid detection of saffron quality is limited. In the next step of this study, the capability of two Vis-NIR and RGB imaging techniques to detect and classify saffron samples was investigated. Applying various machine learning methods using Vis-NIR and RGB image features showed that the RBF neural network can be a suitable algorithm for the defined goal. In addition, comparing the results of NIR and RGB images showed that RGB images had a higher capability. Based on the fourteen features selected from the RGB images, the RBF classifier achieved 99.52% accuracy. Moreover, its generalizability was acceptable. According to the results of this research, this approach can provide a more efficient, cost-effective, and accessible method for detecting counterfeit saffron, which can benefit both consumers and the saffron industry. Therefore, exploring the potential of non-destructive testing methods for saffron authentication can be a promising area of research. In this regard, designing a mobile application can make the results of this research more accessible.

## Figures and Tables

**Table 1:** Used color models, including channels and transformation function from RGB [30].

Color Space	Channel	Transformation from RGB
rgb	R	r = *R*/(*R* + *G* + *B*)
	G	g = *G*/(*R* + *G* + *B*)
	B	b = *B*/(*R* + *G* + *B*)
XYZ	X	X = 0.607*R* + 0.174*G* + 0.200*B*
	Y	Y = 0.299*R* + 0.587*G* + 0.114*B*
	Z	Z = 0.066*G* + 1.116*B*
HSI	H	$H=\{\begin{array}{ll} \cos^{-1}\left\{\frac{0.5\left[\left(R-G\right)+\left(R-B\right)\right]}{{\left[{\left(R-G\right)}^{2}+\left(R-G\right)\left(G-B\right)\right]}^{\frac{1}{2}}}\right\} & if\;B\leq G\\ 360-\cos^{-1}\left\{\frac{0.5\left[\left(R-G\right)+\left(R-B\right)\right]}{{\left[{\left(R-G\right)}^{2}+\left(R-G\right)\left(G-B\right)\right]}^{\frac{1}{2}}}\right\} & if\;B>G \end{array}$
	S	$S=1-\frac{3}{\left(R+G+B\right)}\;[\min\left(R,G,B\right)]\;$
	I	$I=\frac{1}{3}\left(R+G+B\right)$
La*b*	L*	$L^{*}=\left\{116Y^{1/3}\;\;if\;\;Y>k\;;\;\;903.3Y\;if\;\;Y\leq k\right\}\;with\;\;k=0.008856$
	a*	$\begin{array}{cc} a^{*}=500(\;f\left(X\right)\;\;- & f\left(Y\right))\;with\;f\left(t\right)\\ & =\left\{t^{1/3}\;if\;t>k;\;\;7.787t+0.1379\;if\;t\leq k\right\} \end{array}$
	b*	$b^{*}=200\left(f\left(Y\right)-f\left(Z\right)\right)$

**Table 2:** Syntax of various training algorithms.

Training Algorithm	Symbol	Function
Levenberg-Marquardt back propagation	T1	Trainlm
Bayesian regularization	T2	Trainbr
Scaled conjugate gradient backpropagation	T3	Trainscg
Resilient backpropagation (Rprop)	T4	Trainrp
Variable learning rate backpropagation	T5	Traingdx
Gradient descent with momentum backpropagation	T6	Traingdm
gradient descent with adaptive learning rate backpropagation	T7	Traingda
Gradient descent backpropagation	T8	Traingd
BFGS quasi-Newton backpropagation	T9	Trainbfg
Powell–Beale conjugate gradient backpropagation	T10	Traincgb
Fletcher–Powell conjugate gradient backpropagation	T11	Traincgf
Polak–Ribiere conjugate gradient backpropagation	T12	Traincgp
One step secant backpropagation	T13	Trainoss

**Table 3:** Accuracy values of different classifiers in genuine and fake saffron classification based on the characteristics of NIR and RGB images.

	RGB Features			NIR Features		
Classifiers	Train	Test	Total	Train	Test	Total
RBF	99.70	98.81	99.52	94.78	94.82	94.79
MLP	99.78	97.32	99.29	89.66	89.66	89.58
KNN	100	88.10	97.62	100	79.31	95.83
SVM	100	71.43	94.29	99.13	75.86	94.44
SOM	89.29	89.29	89.29	80	72.41	78.47
LVQ	52.68	52.38	52.62	43.48	40	43.4

**Table 4:** The value of RBF classifier accuracy for seven fake and genuine classes using Vis-NIR spectral features.

	F1	F2	F3	F4	G1	G3	G2	All
Train	100	96.87	87.5	90.62	90.62	97.14	100	94.78
Test	100	100	87.5	87.5	87.5	100	100	94.82
Total	100	97.5	87.5	90	90	97.72	100	94.79

**Table 5:** The results of the RBF neural network generalizability based on Vis-NIR spectral features.

Training Size (%)	Train	Test	Total
80	94.78	94.82	94.79
70	95.05	93.02	94.44
60	94.19	93.1	93.75
50	93.75	90.97	92.36

**Table 6:** Evaluation of RBF classifier accuracy values for seven defined saffron classes based on different features of RGB images.

Features	Train	Test	Total
All	99.70	98.81	99.52
Slected (14 features)	99.70	98.81	99.52
HSI	99.11	98.81	99.05
RGB	92.26	91.76	92.14
L*a*b*	89.88	92.86	90.48
HSIstd	88.39	88.1	88.33
HSIave	88.39	83.33	87.38
RGBave	82.14	85.71	82.86
L*a*b*ave	79.46	89.29	81.43
RGBstd	77.98	82.14	78.81
L*a*b*std	69.94	70.24	70.00
texture	62.50	65.48	63.10

**Table 7:** The accuracy of the RBF classifier for seven saffron classes based on selected features of RGB images.

	F1	F2	F3	F4	G1	G3	G2	All
Train	100	100	97.92	100	100	100	100	99.70
Test	100	100	91.67	100	100	100	100	98.81
Total	100	100	96.67	100	100	100	100	99.52

**Table 8:** Generalizability results for the RBF classifier based on selected features of RGB images.

Training Size (%)	Train	Test	Total
80	99.70	98.81	99.52
70	99.66	98.41	99.29
60	99.21	98.25	98.83
50	98.57	97.62	98.10

**Table 9:** Similar research results.

Method	Objective	Base on Method	Accuracy	Reference
Near-infrared spectroscopy	Determination of crocin	Destructive	93.4–96.3%	[20]
Computer vision	Saffron color quality characterization	Non-destructive	99%	[13]
Deep Learning	Detection of Saffron Adulteration	Non-destructive	99.8%	[15]
Electronic nose	Detection of Saffron Adulteration	Destructive	86.87–100%	[48]
Electronic tongue	Detection of Saffron Adulteration	Destructive	86.21–96.15%	[49]
Near-infrared spectroscopy and chemometrics	Detection of Saffron Adulteration	Destructive	99%	[21]
Matrix-assisted laser desorption ionization mass spectrometry (MALDI-MS/MS)	Detection of Saffron Adulteration	Destructive	99%	[50]
Proposed method	Genuine and fake saffron classification	Non-destructive	99.52%	Our method

## Data Availability

The data presented in this study are available on request from the corresponding author. The data are not publicly available due to privacy.

## Abbreviations

**Nomenclature**	**Definition**
F1	Dyed citrus blossom
F2	Safflower
F3	Mixed stamen and dyed straw
F4	Fiber
G1	Microwave-dried saffron
G2	Freeze-dried saffron and:
G3	Hot-air-dried saffron
RGB	Red, green, and blue color components, respectively
HSI	Hue, saturation, and intensity color components, respectively
L*a*b*	Luminance, the red-green axis, and the blue-yellow axis color components, respectively
RBF	Radial Basis Function
MLP	Multilayer Perceptron
KNN	K-nearest neighbor
SVM	Support vector machine
SOM	Self-organized map
LVQ	Learning vector quantization
